# Programmed Frozen Embryo Transfer in the Absence of Corpus Luteum: Impact on Maternal Cardiovascular Adaptation, Endothelial Function and Risk of Preeclampsia

**DOI:** 10.3390/biology15201834

**Published:** 2026-10-13

**Authors:** Angeliki Gerede, Foteini Gkaitatzi, Efthymios Oikonomou, Panayiota Papasozomenou, Vasiliki Kourti, Nektaria Zagorianakou, Panagiotis Tsikouras, Nikoletta Koutlaki, Menelaos Zafrakas, Ekaterini Domali

**Affiliations:** 1 Department of Obstetrics and Gynecology, Democritus University of Thrace, 69100 Alexandroupolis, Greece; gkaitatzifoteini@hotmail.com (F.G.); efthymiosoikonomou@gmail.com (E.O.); ptsikour@med.duth.gr (P.T.); nkoutlak@med.duth.gr (N.K.); 2 School of Health Science, International Hellenic University, 57400 Thessaloniki, Greece; papasozomenou248@gmail.com; 3 Department of Nursing, School of Health Sciences, University of Ioannina, 45500 Ioannina, Greece; zagorianakou@uoi.gr; 4 First Department of Obstetrics and Gynecology, Alexandra Hospital, Medical School, National and Kapodistrian University of Athens, 11528 Athens, Greece; kdomali@yahoo.fr

**Keywords:** corpus luteum, programmed frozen embryo transfer, maternal cardiovascular adaptation, hypertensive disorders of pregnancy, preeclampsia

## Abstract

Programmed frozen embryo transfer (FET) cycles are an integral part of assisted reproductive technologies (ARTs). They rely exclusively on the administration of exogenous estrogens and progesterone to prepare the endometrium, and they are characterized by the absence of a functional corpus luteum (CL). Pregnancies resulting from FET cycles have been associated with an increased risk of complications related to placental dysfunction, such as preeclampsia and fetal growth restriction (FGR). Besides progesterone production, the CL secretes vasoactive and angiogenic factors essential for the regulatory mechanisms that occur during the early stages of pregnancy. Its absence results in a different endocrine environment, which may be associated with differences in normal maternal cardiovascular remodeling. In pregnant women without CL function, reduced vasodilation, increased systemic vascular resistance, altered cardiac output, and inadequate renal adaptation have been reported. In addition, disturbances in angiogenic balance and trophoblast invasion may contribute to abnormal placentation and the development of preeclampsia. Accordingly, the absence of the CL in programmed FET cycles may represent a critical endocrine disorder, predisposing patients to maternal cardiovascular disturbance and adverse obstetric outcomes. Recognition of this potential endocrine and vascular interaction has important implications for cycle selection, risk stratification, and preventive strategies in the implementation of assisted reproduction protocols.

## Simple Summary

Programmed frozen embryo transfer cycles are characterized by the absence of a functional corpus luteum. Besides progesterone production, the corpus luteum secretes vasoactive and angiogenic factors essential for the regulatory mechanisms that occur during the early stages of pregnancy, and its absence results in a different endocrine environment that may be associated with differences in normal maternal cardiovascular remodeling. Pregnancies resulting from frozen embryo transfer cycles have been associated with an increased risk of complications related to placental dysfunction. The present review provides a comprehensive overview of the mechanisms underlying the absence of the luteal phase and its contribution to maternal cardiovascular adaptation and the increased risk of pregnancy complications, thereby paving the way for the development of new approaches aimed at minimizing the risks associated with FET.

## 1. Introduction

The “freeze-all” strategy has gained ground in recent years due to its promising efficacy, improved live birth rates, and reduced risk of ovarian hyperstimulation syndrome (OHSS) [1,2,3]. In the USA, the use of FET increased from 7.9% of all embryo transfers in 2004 to 40.7% in 2013, and similar trends have been observed globally [4]. However, an increased incidence of hypertensive disorders of pregnancy (HDPs) has been observed after programmed FET, with the underlying mechanisms remaining unclear [5].

During transfer of thawed embryos, there are two options for preparing the endometrium to achieve an optimal state for implantation: (a) in natural and stimulated cycles, detection of ovulation determines the timing of embryo thawing and transfer; (b) in programmed cycles, hormonal replacement is used to establish endometrial receptivity for embryo implantation, providing flexibility in scheduling [5]. Existing evidence comparing different regimens for endometrial preparation has not shown significant differences in implantation, pregnancy, or live birth rates after FET [6]. However, recent observational studies [7,8] have reported a higher risk of HDPs and reduced aortic adaptation among women undergoing programmed FET cycles compared with natural or stimulated regimens, fresh cycles, or spontaneous conception, with preeclampsia (PE) occurring in 12.8% vs. 3.9% (*p* = 0.02) and severe PE in 9.6% vs. 0.8% (*p* = 0.002) [4]. Despite the replacement of luteal support with exogenous estrogen and progesterone in programmed cycles, the absence of the corpus luteum (CL) due to suppressed follicular development may predispose patients to adverse obstetric outcomes, such as PE [9], and the lack of vasoactive agents produced by the CL after ovulation in a natural cycle may impact maternal vascular health and subsequent placental development [10,11,12].

Programmed FET differs biologically from natural and modified-natural FET, because it relies on exogenous hormonal supplementation without spontaneous ovulation and corpus luteum formation. Consequently, the endocrine environment during early pregnancy differs between these protocols, as corpus luteum-derived factors involved in maternal vascular adaptation and placental development are absent in programmed cycles. This biological distinction may contribute to differences in pregnancy outcomes, including hypertensive disorders and preeclampsia, although the underlying mechanisms remain incompletely understood [5,6].

The present narrative review aims to provide a comprehensive overview of the mechanisms underlying the absence of the luteal phase and its contribution to maternal cardiovascular adaptation and the increased risk of PE, thereby paving the way for the development of new approaches aiming at minimizing the risks associated with FET.

## 2. Literature Search Strategy

A comprehensive literature search was conducted using the electronic databases PubMed and ScienceDirect to identify relevant studies pertaining to the research question. The search strategy included combinations of keywords and terms related to frozen embryo transfer (“frozen embryo transfer”, “FET”, “frozen-thawed embryo transfer”), FET protocols (“programmed FET”, “artificial cycle”, “hormone replacement”, “natural FET”, “modified-natural FET”), corpus luteum (“corpus luteum”, “luteal function”, “luteal phase”), and hypertensive pregnancy outcomes (“preeclampsia”, “pre-eclampsia”, “hypertensive disorders of pregnancy”, “HDP”). No restrictions were imposed on the publication date in order to ensure a comprehensive search of the available literature. Studies published in English were considered eligible for inclusion. The exclusion criteria comprised articles published in languages other than English and review articles. The retrieved records were subsequently screened against the predefined eligibility criteria, and studies meeting the inclusion criteria were selected for further analysis.

## 3. Corpus Luteum Function and Underlying Mechanisms

The CL is a transient endocrine organ that secretes a diverse repertoire of hormones and vasoactive mediators in addition to progesterone and estradiol. These include relaxin, vascular endothelial growth factor (VEGF), prorenin, renin, inhibin A, and several locally acting cytokines and angiogenic factors [7]. While exogenous estrogen and progesterone administered during programmed FET adequately support endometrial receptivity and pregnancy maintenance, they do not replace the other non-steroidal secretory products. Consequently, programmed FET pregnancies may begin in a unique endocrine environment characterized by the absence of circulating luteal vasoactive hormones. This endocrine deficiency is increasingly recognized as a plausible contributor to impaired maternal cardiovascular adaptation and abnormal placentation [11,12].

Furthermore, the absence of the CL [11] in programmed FET cycles due to ovulation suppression alters the hormonal environment and may lead to a lack of circulating vasoactive proteins and hormones, such as relaxin [10,12,13] and VEGF; this may contribute to reduced arterial compliance in early pregnancy and consequently to an increased risk of HDPs [9] and PE. Relaxin is a circulating 6 kD peptide; in animal studies, it has been shown to cause systemic and renal vasodilation, which is partly due to the production of nitric oxide (NO) [14,15,16]. Relaxin promotes systemic vasodilation by stimulating endothelial NO-synthase through activation of endothelin type-B receptors, resulting in increased NO-bioavailability, enhanced arterial compliance, reduced systemic vascular resistance, and increased renal plasma flow [8]. These physiological adaptations occur during the first trimester and are fundamental for accommodating the expanding maternal circulation. In pregnancies conceived without a CL, circulating relaxin levels are nearly undetectable, and prospective studies have demonstrated attenuation of the normal increase in cardiac output together with reduced arterial compliance. Although placental vasodilators may be partially compensated later in pregnancy, impaired cardiovascular adaptation during early gestation may create conditions that predispose patients to hypertensive disorders [11].

NO may also contribute to endothelial homeostasis by promoting the mobilization and migration of endothelial progenitor cells (EPCs), thereby supporting endothelial repair and vascular adaptation during pregnancy [17]. Circulating progenitor cells (CPCs), including EPCs, are considered important for maintaining endothelial integrity and facilitating vascular repair. Consistent with their potential role in maternal vascular health, reduced numbers and impaired function of CPCs and late-outgrowth endothelial progenitor cells have been reported in both maternal and cord blood from pregnancies complicated by pre-eclampsia (PE) [18,19,20]. These findings suggest that alterations in NO-dependent endothelial repair mechanisms and progenitor-cell function may contribute to the impaired endothelial adaptation characteristic of PE.

The CL also contributes to physiological activation of the renin–angiotensin–aldosterone system (RAAS) through secretion of renin and prorenin during early pregnancy. Controlled activation of the RAAS facilitates sodium retention, plasma volume expansion, and the maintenance of adequate uteroplacental perfusion despite systemic vasodilation. In programmed FET cycles lacking a CL, this endogenous luteal contribution is absent, potentially leading to suboptimal plasma volume expansion and impaired circulatory adaptation. Although direct human evidence remains limited, insufficient plasma volume expansion has long been recognized as a characteristic feature of pregnancies that subsequently develop PE, making altered RAAS activation a biologically plausible mechanism linking CL absence with disease development [21].

Relaxin also appears to inhibit the increase in intracellular calcium promoted by angiotensin II in cultured rat mesangial cells independently of NO and angiotensin II receptor type 2 (AGTR2) [22]. The same has been observed in human mesangial cells, which may contribute to the increase in glomerular filtration rate (GFR), possibly through increasing the filtration surface during normal pregnancy [23]. In a study by Sarwar et al. [24], relaxin did not follow the traditional dose-response relationship, with increases in cyclic adenosine monophosphate (cAMP) and cyclic guanosine monophosphate (cGMP) levels being dependent on the relaxin dose and cell type, suggesting that an optimal intermediate concentration is required for a healthy pregnancy and normal vascular response.

Regarding cardiovascular adaptation, a normal pregnancy is characterized by adaptive changes in the cardiovascular system, including a decrease in mean arterial pressure (MAP), an increase in cardiac output (CO), and a decrease in systemic vascular resistance, even before the full establishment of the maternal-fetal-placental unit, as early as 6 weeks [7]. The absence of CL during the first trimester has been associated with an attenuation of the expected decrease in carotid-femoral pulse wave velocity, an increase in carotid-femoral transit time during the first trimester [8] and an insufficient increase in central arterial compliance (CAC) [25]. Furthermore, the above condition has also been associated with higher levels of creatinine, sodium, and total carbon dioxide in early pregnancy [12]. As for relaxin, it has been shown to be critical in changes in maternal circulation during early and mid-gestation in pregnant rat models [10,11,14], and it may contribute to the increase in GFR and the decrease of plasma osmolarity during the first trimester in humans [16]; in contrast, its deficiency during this stage may impair maternal vascular adaptations, affecting both renal and placental function [7,23].

In addition to the direct effects of relaxin on the cardiovascular system, an indirect role mediated by the endometrium in the pathogenesis of the disease has been proposed [26]. In vitro studies suggest that relaxin plays a key role in placentation by regulating endometrial remodeling and angiogenesis [27]. Dysregulated endometrial maturation may affect early trophoblast development and invasion, which have been implicated in a range of pregnancy complications, from PE to placenta accreta. It is possible that exposure of trophoblast cells to relaxin and other factors derived from the CL activates multiple molecular pathways involved in normal placental development. Interestingly, the expression of endometrial relaxin increases during pregnancy, and relaxin induces decidualization, as it has been shown to increase the secretion of factors and markers such as prolactin and insulin-like growth factor binding protein-1 (IGFBP-1) in synergy with methoxyprogesterone acetate, VEGF, and the mRNA and protein of glycodelin [23]. An overview of proposed mechanisms linking CL absence in programmed FET cycles to hypertensive disorders of pregnancy is presented in Table 1.

## 4. Hypertensive Disorders of Pregnancy and Preeclampsia Risk in Programmed FET Cycles

HDPs are the second leading cause of maternal and neonatal morbidity worldwide [28] and one of the leading causes of maternal death in the United States [29]. The rate of HDP diagnoses doubled from 6.0% to 12.0% between 2000 and 2018, in part due to a threefold increase in the rate of deliveries with risk factors for HDPs, rising from 9.6% to 24.6% [30]. Preeclampsia (PE), defined as the onset of hypertension after 20 weeks of gestation, in association with significant proteinuria and/or evidence of organ damage, is a significant cause of maternal and fetal morbidity and mortality worldwide, with an estimated global incidence of 4.6% [31,32]. As for pregnancies after the use of ART, it has been shown that they are associated with a slightly higher risk of maternal and neonatal complications [33]; factors specific to fertility treatments, including the type of ART procedure, egg and sperm handling, gonadotropin dose and use of donor eggs, appear to contribute more to the risk of PE than older maternal age, body mass index (BMI), multifetal pregnancies and pre-existing maternal diseases [7]. Pregnancies resulting from FET are characterized by the absence of CL and are associated with an almost two-fold risk of developing HDPs compared with pregnancies in the presence of CL, either after spontaneous conception or in modified-natural FET cycles [4,31,34]. Several studies have shown an increased risk of HDPs in singleton pregnancies after programmed FET cycles compared with natural, modified-natural and stimulated FET cycles [4,8,30,35,36,37,38,39,40,41,42,43,44,45,46,47,48,49,50,51,52,53,54,55,56,57,58,59]. Makhijani et al. reported that, after adjustment for age, BMI, diagnosis, smoking status, history of diabetes mellitus or hypertension, primary infertility diagnosis, number of embryos transferred, and use of PGT, women undergoing programmed FET had approximately 2.4-fold higher odds of developing HDPs than those undergoing natural FET (adjusted OR 2.39; 95% CI 1.37–4.17; *p* < 0.01) [37].

The reason for the higher risk of HDPs in programmed FET cycles remains unknown. Although initially thought to be a result of the freeze–thaw process, it has been shown that programmed FET cycles were three times more likely to result in PE compared with modified-natural FET cycles, where vitrified embryos were transferred to an unstimulated ovulation cycle in the presence of only one CL [8]. Recently, the influence of embryo stage on obstetric complications and perinatal outcomes following programmed compared to natural FET cycles has been the subject of a systematic review and meta-analysis [60]. Several factors secreted by the CL have been shown to play a critical role in the transformation of the maternal cardiovascular system during early pregnancy. In the prospective study by Von Versen-Hoynck et al. [7], pregnancies without CL during the first trimester were associated with an increased risk of PE, while the expected increases in CO, left atrial size, peak left ventricular filling velocity in early diastole (E-wave velocity), peripheral-to-central pulse pressure ratio, total arterial compliance (AC) and reduction in growth index were significantly attenuated or absent. Specifically, women with 0 or >3 CL did not experience a decrease in MAP compared with those with 1 CL (*p* = 0.05), the reactive hyperemia index (RHI) was reduced in women with 0 CL compared with 1 CL (*p* = 0.04), while the baseline pulse wave amplitude was higher with >3 CL compared with 1 CL (*p* = 0.01) or 0 CL (*p* = 0.01). In the same study, in cycles involving only FET, a lower RHI and a higher augmentation index (AI) were observed in FETs without CL compared with FETs in a natural cycle (*p* = 0.03). In addition, the number of angiogenic and non-angiogenic CPCs was lower. Subsequently, when the placenta became the main source of these factors, cardiovascular measurements showed recovery in the no-CL group, except for E-wave velocity, which remained reduced [24]. The finding that vascular dysfunction due to CL deficiency precedes the clinical onset of PE and is considered part of its pathogenesis is supported by other studies; in a recent study, MAP measured between 11 and 14 weeks of gestation was a strong predictor for the development of gestational hypertension and PE [61]. Endothelial dysfunction as evidenced by lower RHI was also found in a group of 105 preeclamptic women compared with 110 normotensive, pregnant controls at 30 to 32 weeks of gestation [7]. Women with pre-eclampsia demonstrated significantly lower RHI values [1.70 (1.04–3.61) vs. 1.81 (1.18–4.62) au; *p* < 0.05] and log-transformed RHI values [0.31 (−0.03–1.24) vs. 0.48 (0.00–1.87) au; *p* < 0.01] compared with normotensive controls [7].

Several other studies that assessed flow-induced dilation have shown worse vascular function as early as the second trimester in women who subsequently developed PE [62,63]. Improper adaptation of the circulatory system during early pregnancy has been associated with adverse pregnancy outcomes. For example, in the retrospective cohort study by Wang et al. [5], an increased risk of PE and postpartum hemorrhage (PPH) was noted in women with singleton delivery after programmed FET, possibly related to the absence of the CL. This study confirmed that programmed FET is an independent risk factor, while the incidence of gestational diabetes mellitus (GDM), placenta previa (PP), placental abruption (PA), macrosomia, large for gestational age (LGA), and small for gestational age (SGA) were similar between programmed and natural cycles (NCs). Gu et al. demonstrated that pregnancies following programmed FET were associated with a higher risk of HDPs in ovulatory women with the pretreatment cycle of hormone replacement therapy, with gonadotropin-releasing hormone analogue carrying the highest risk of PE [64].

In contrast, Rodriguez et al. included 994 patients, of whom 751 underwent natural FET and 243 underwent programmed FET. The two groups were comparable in terms of age (mean 37 years), race, and ethnicity. However, women in the programmed FET group had a significantly higher obstetric comorbidity index (median difference, *p* = 0.022), indicating a marginally greater baseline burden of chronic disease. Median BMI was also significantly higher in the programmed FET group than in the natural FET group [31 (IQR 28–36) vs. 30 (IQR 27–34) kg/m^2^, *p* = 0.013]. Although gestational hypertension or pre-eclampsia occurred more frequently among women undergoing programmed FET than among those undergoing natural FET (24% vs. 19%), this difference was not statistically significant (*p* = 0.12) [65]; therefore, factors such as body mass index (BMI) and comorbidities, which influence pregnancy outcomes, should be taken into account in risk stratification and counseling. Likewise, in the study by Dallagiovanna et al. [43], women who received hormone replacement therapy had a risk of developing gestational hypertension and PE similar to those who followed a natural cycle, in low-risk singleton pregnancies. Programmed FET cycles were associated with a significantly higher risk of gestational hypertension than natural cycles (OR 1.40, 95% CI 1.15–1.70, *p* = 0.0006, I^2^ = 23%). In subgroup analyses, this association remained significant among women undergoing blastocyst transfer (OR 1.87, 95% CI 1.27–2.75, *p* = 0.002, I^2^ = 25%), whereas no significant difference was observed following cleavage-stage embryo transfer (OR 0.85, 95% CI 0.48–1.51, *p* = 0.59, I^2^ = 0%) [60].

Similarly, programmed cycles were associated with a significantly increased risk of preeclampsia compared with natural cycles (OR 2.15, 95% CI 1.94–2.39, *p* < 0.00001, I^2^ = 0%). This association was also observed in the blastocyst-transfer subgroup (OR 2.23, 95% CI 1.93–2.56, *p* < 0.00001, I^2^ = 0%). In contrast, the single study assessing cleavage-stage embryo transfer found no significant difference in the risk of preeclampsia between programmed and natural cycles (OR 1.19, 95% CI 0.58–2.42, *p* = 0.64; I^2^ not applicable) [60].

## 5. Biomarkers of Maternal Adaptation and Preeclampsia Risk in Programmed Frozen Embryo Transfer

The absence of a functional corpus luteum (CL) in programmed FET cycles creates a distinctive endocrine environment characterized by reduced exposure to several luteal-derived vasoactive and angiogenic mediators that may contribute to maternal cardiovascular adaptation and pre-eclampsia (PE) risk [30]. Biomarker research in this setting should therefore distinguish between established biomarkers of PE and candidate biomarkers that may be particularly relevant to programmed FET and CL deficiency. Among the most extensively validated biomarkers of PE are the angiogenic factors soluble fms-like tyrosine kinase-1 (sFlt-1) and placental growth factor (PlGF), which reflect the imbalance between anti-angiogenic and pro-angiogenic signaling associated with placental dysfunction [66]. Increased circulating sFlt-1, reduced PlGF concentrations, and an elevated sFlt-1/PlGF ratio are consistently associated with PE, and the sFlt-1/PlGF ratio has demonstrated clinical utility, particularly in women presenting with suspected PE and for the assessment of short-term risk [67]. However, evidence regarding differences in these established angiogenic biomarkers according to FET protocol remains limited. Whether CL deficiency in programmed FET is associated with an altered angiogenic profile, and whether this contributes to the increased risk of PE observed in this population, therefore remains an area for further investigation [68].

In contrast, luteal-derived hormones and markers of maternal cardiovascular adaptation should currently be regarded as candidate biomarkers in the context of programmed FET rather than established PE biomarkers. Relaxin is of particular interest because it is predominantly produced by the CL during early pregnancy and contributes to physiological cardiovascular and renal adaptations through pathways involving endothelial nitric oxide signaling and vascular compliance [69]. The markedly reduced circulating relaxin concentrations observed in pregnancies following programmed FET may therefore provide a potential mechanistic link between CL absence, impaired maternal vascular adaptation, and hypertensive disorders of pregnancy [30]. Nevertheless, evidence supporting relaxin as a predictive biomarker for PE remains insufficient for clinical application. Similarly, other markers of endothelial dysfunction, including endothelin-1, asymmetric dimethylarginine (ADMA), symmetric dimethylarginine (SDMA), inflammatory mediators, and markers of oxidative stress, may provide complementary information on vascular maladaptation but remain investigational in programmed FET populations [70]. Future studies should determine whether these candidate markers provide additional predictive information beyond established PE biomarkers and whether their combination with angiogenic and luteal-derived markers can improve risk stratification, specifically among women undergoing programmed FET [30].

An overview of potential biomarkers reflecting altered maternal adaptation and PE risk in CL-deficient programmed FET cycles is presented in Table 2.

## 6. Cardiovascular Imaging and Functional Assessment in Corpus Luteum-Deficient Pregnancies

The potential contribution of the corpus luteum (CL) to maternal cardiovascular adaptation provides a mechanistic framework for understanding the cardiovascular phenotype observed after programmed FET. Normal pregnancy involves substantial cardiovascular remodeling, including increased cardiac output and plasma volume, reduced systemic vascular resistance, and increased arterial compliance [71]. In addition to estradiol and progesterone, the CL produces vasoactive mediators, particularly relaxin, which may promote these adaptations through effects on endothelial function, nitric oxide signaling, vascular compliance, and renal hemodynamics [30]. In programmed FET, the absence of a functional CL has been associated with attenuated increases in arterial compliance, suggesting an impaired vascular adaptation that may precede the clinical onset of PE [8]. Cardiovascular maladaptation may therefore represent an intermediate phenotype linking CL deficiency to hypertensive pregnancy complications.

Cardiovascular imaging and functional assessment may complement circulating biomarkers in characterizing this phenotype. Echocardiography can detect alterations in left ventricular geometry, diastolic function, and myocardial deformation, including global longitudinal strain, while pulse wave velocity and flow-mediated dilation provide measures of arterial stiffness and endothelial function [72,73]. Integrating these measures with circulating biomarkers may provide a more comprehensive assessment of the consequences of CL absence. In particular, combining cardiovascular measures with CL-derived candidates such as relaxin and established angiogenic biomarkers such as sFlt-1 and PlGF may help distinguish impaired maternal cardiovascular adaptation from subsequent placental dysfunction. However, evidence for such an integrated approach in programmed FET remains limited, and prospective longitudinal studies are needed to establish the predictive value of these measures for PE [30].

## 7. Clinical Implications and Future Directions

The clinical implications of CL deficiency should be considered in the context of individualized FET protocol selection. In women with ovulatory cycles, natural or modified-natural FET preserves endogenous CL function and has been associated with a lower incidence of hypertensive disorders of pregnancy than programmed FET, while reproductive outcomes may be comparable in appropriately selected populations [36,74]. However, protocol selection is influenced by clinical and reproductive characteristics, including ovulatory status, menstrual regularity, PCOS or anovulation, previous treatment history, and clinical practice. Differences in obstetric outcomes between protocols may therefore reflect both CL status and differences in the underlying populations.

In programmed FET, exogenous estradiol and progesterone provide the principal hormonal requirements for endometrial preparation and early pregnancy maintenance but do not fully reproduce the endocrine function of the CL. Consequently, several CL-derived vasoactive and angiogenic mediators remain absent or substantially reduced [25]. Relaxin is of particular interest because it is predominantly produced by the CL during early pregnancy, whereas estradiol and progesterone are subsequently also produced by the placenta. In the study by von Versen-Höynck et al. [7], relaxin showed the strongest association with the vascular parameters examined, supporting further investigation of CL-derived pathways in maternal cardiovascular adaptation. However, these findings remain mechanistic and hypothesis-generating.

Accordingly, relaxin replacement should be considered an investigational strategy rather than an established therapy. Its efficacy, optimal timing and dose, and maternal and fetal safety remain to be determined [23]. Likewise, although relaxin is a biologically plausible candidate biomarker, its predictive performance for PE remains insufficient for clinical application [27]. Future studies should evaluate whether integrated models incorporating CL-derived mediators, established angiogenic biomarkers, and longitudinal cardiovascular measures can improve risk stratification after programmed FET. Such studies may also help determine whether the increased risk of PE reflects CL absence itself, differences in the women selected for different FET protocols, or both.

## 8. Confounding and Selection Bias

The findings should be interpreted in light of potential confounding factors within the available evidence. Women undergoing programmed FET may differ from those undergoing natural or modified-natural FET in several characteristics that independently influence maternal cardiovascular adaptation and the risk of pre-eclampsia. Maternal age, BMI, parity, previous obstetric history, PCOS or anovulation, and the underlying diagnosis of infertility are particularly relevant. PCOS and anovulation may be especially important, as programmed FET is commonly used in women with absent or irregular ovulation, while these conditions may themselves be associated with metabolic and vascular abnormalities and an increased risk of adverse pregnancy outcomes. Similarly, differences in infertility diagnoses and parity between treatment groups may contribute to differences in baseline cardiovascular and obstetric risk. If these factors are not consistently reported or adequately adjusted for, residual confounding may partly account for the associations observed between programmed FET, corpus luteum absence, and pre-eclampsia.

Selection bias is another important consideration, as the choice of FET protocol is generally influenced by clinical characteristics rather than being randomly assigned. Women selected for programmed FET may therefore represent a clinically distinct population, particularly with respect to ovulatory function, menstrual regularity, PCOS, previous treatment history, and clinician or center preference. Differences in study eligibility criteria and patient populations may further contribute to heterogeneity across the literature. Consequently, the observed association between the absence of a corpus luteum and maternal cardiovascular or hypertensive outcomes should not necessarily be interpreted as a direct causal effect. Greater consistency in the reporting and adjustment of key maternal and reproductive characteristics, including age, BMI, PCOS/anovulation, infertility diagnosis, parity, and previous pre-eclampsia, would strengthen future evidence and help to distinguish the effect of corpus luteum absence from differences in the underlying characteristics of women undergoing different FET protocols.

Furthermore, embryo stage at transfer, embryo quality, and other embryo characteristics may be associated with implantation dynamics and placentation and could therefore contribute to differences in pregnancy outcomes between FET protocols. Similarly, the use of donor oocytes may represent an important consideration, as donor status is associated with distinct maternal–fetal immunological and obstetric characteristics that may independently influence the risk of PE. Therefore, the increased risk observed in donor oocyte pregnancies should not necessarily be attributed solely to the absence of the corpus luteum, as donor status itself may represent an independent contributor to adverse pregnancy outcomes. Consequently, differences in embryo characteristics and donor status between study groups may partly account for, or modify, the observed association between FET protocol and PE. These factors should therefore be considered when interpreting the available evidence, and future studies should appropriately adjust for them in multivariable analyses to better delineate the independent association between FET protocol and PE risk.

## 9. Remaining Knowledge Gaps

Although the association between programmed FET without a CL and PE is now supported by multiple cohort studies and systematic reviews, several mechanistic questions remain unanswered. The relative contribution of relaxin deficiency, altered RAAS activation, impaired decidualization, abnormal angiogenic signaling, and immune dysregulation have not been fully elucidated. Furthermore, not all women undergoing programmed FET develop HDPs, suggesting that maternal cardiovascular reserve, genetic susceptibility, metabolic status, and pre-existing endothelial function likely modify individual risk. Future studies integrating cardiovascular phenotyping, angiogenic biomarkers, endocrine profiling, and placental molecular analyses will be essential to establish causal pathways and identify preventive therapeutic strategies [31,33].

## 10. Conclusions

FET improves flexibility in fertility preservation and genetic testing. Although it is safe and effective compared with fresh ET, programmed FET cycles in the absence of CL have been associated with impaired cardiovascular adaptation, decidualization and placental formation. However, artificial cycles offer the ideal background to investigate the potential role of substances secreted by the CL and relaxin in the pathophysiological adaptive changes during early pregnancy. Typically, vascular adaptation depends on the maternal hormonal environment, which is altered in programmed FET cycles due to exogenous hormone administration and the absence of a functional CL, rather than as a consequence of cryopreservation or the fertilization method. This may increase the risk of HDPs, especially in women with additional risk factors and comorbidities. The underlying mechanisms remain unclear, and further research is needed to assess the role of CL-derived factors and to improve personalized treatment approaches. Furthermore, it remains unclear whether FET with ART is associated with early- or late-onset PE, and further clarification may help elucidate underlying mechanisms. It will be important for future studies to consider the protocols used for FET when determining the risk of PE and if relaxin is indeed an important mediator of cardiovascular adaptation during early pregnancy; a possible scenario would be that supplementing relaxin in fertility treatment protocols could potentially improve vascular function. Ideally, in women of reproductive age, FET should be performed in a natural or modified-natural cycle, as this preserves the presence of CL and supports normal maternal adaptation to pregnancy. It should also be taken into account that the lack of other factors derived from the CL besides relaxin may contribute to an increased risk of PE. With the help of transcriptome analysis, bioinformatics and other techniques, biomarkers and molecular mechanisms associated with endometrial receptivity and PE risk can be identified, helping to personalize hormonal supplementation and protocol selection. After decades of focusing on achieving pregnancy, the challenge is now to ensure safer outcomes, with personalized FET protocol selection being a key step.

## Figures and Tables

**Table 1:** Proposed mechanisms linking CL absence in programmed FET cycles to hypertensive disorders of pregnancy.

Molecule/ Factor	Principal Source	Physiological Function	Evidence for Corpus Luteum (CL) Contribution	Evidence in Programmed FET	Proposed Consequence of CL Absence
Relaxin	Corpus Luteum	Promotes systemic and renal vasodilation, increases arterial compliance and glomerular filtration, and may contribute to decidualization and placental development.	The CL is a major source of circulating relaxin during early pregnancy, with markedly lower circulating concentrations in the absence of ovulation.	Programmed FET is characterized by the absence of CL-derived relaxin; observational and mechanistic evidence has linked this endocrine difference with altered maternal cardiovascular adaptation.	Reduced relaxin exposure may influence maternal cardiovascular and renal adaptation and may contribute to altered placentation and an increased risk of HDPs, although causality remains uncertain.
Nitric oxide (NO)	Endothelium; downstream of several vasoactive pathways, including relaxin signaling	Promotes endothelial function, vasodilation, and vascular remodeling.	Relaxin can stimulate NO-mediated vasodilation, suggesting an indirect contribution of CL-derived relaxin to vascular adaptation.	The absence of CL-derived relaxin in programmed FET may reduce this physiological signaling pathway, although direct evidence regarding NO bioavailability in programmed FET remains limited.	Altered NO-mediated vascular signaling may influence maternal vascular adaptation and endothelial function.
Vascular endothelial growth factor (VEGF)	Corpus luteum and reproductive/placental tissues	Regulates angiogenesis and vascular remodeling involved in implantation and placental development.	The CL produces VEGF, which contributes to luteal angiogenesis and vascular function; VEGF is also produced by placental and decidual tissues.	Direct evidence linking the absence of CL-derived VEGF specifically to programmed FET outcomes is limited.	Alterations in luteal VEGF signaling may influence early vascular adaptation and placentation, potentially contributing to differences in hypertensive pregnancy outcome.
Renin–angiotensin–aldosterone system (RAAS)	Corpus luteum, kidney, vascular and placental tissues	Regulates vascular tone, sodium and water balance, plasma-volume expansion, and uteroplacental perfusion.	The CL expresses components of the renin–angiotensin system, including renin and prorenin, which may contribute to reproductive and early pregnancy physiology.	Programmed FET lacks the ovarian contribution associated with spontaneous ovulation and CL formation; however, the extent to which this alters systemic RAAS activity remains incompletely established.	Differences in RAAS signaling may influence plasma-volume expansion and maternal cardiovascular adaptation, potentially contributing to susceptibility to HDPs and PE.

**Table 2:** Potential biomarkers reflecting altered maternal adaptation and PE risk in CL-deficient programmed FET cycles.

Biomarker	Biological Role	Potential Relevance in Programmed FET Cycles	Evidence
sFlt-1	Anti-angiogenic factor reflecting placental endothelial dysfunction. Increased levels precede clinical PE.	May identify enhanced anti-angiogenic signaling associated with increased PE risk after CL-deficient cycles.	Evidence on whether this established angiogenic biomarker differs according to FET protocol remains limited.
PlGF	Pro-angiogenic factor essential for placental vascular development and endothelial function. Reduced levels are associated with PE.	May reflect impaired angiogenic balance resulting from abnormal placental adaptation.	Direct evidence regarding differences in this established angiogenic biomarker according to FET protocol remains limited.
sFlt-1/PlGF ratio	Integrated marker of angiogenic imbalance with established predictive value for PE and adverse outcomes.	Potential tool to investigate whether programmed FET pregnancies exhibit altered angiogenic profiles compared with ovulatory FET cycles.	Direct evidence supporting differences in these established angiogenic biomarkers according to FET protocol remains limited and inconclusive.
Relaxin	CL-derived hormone involved in vasodilation, NO signaling, renal adaptation, and maternal cardiovascular remodeling.	Reduced levels in programmed FET may indicate impaired maternal cardiovascular adaptation and increased susceptibility to HDPs.	Current evidence supporting the use of relaxin as a predictive biomarker for preeclampsia remains insufficient to support its clinical application.
Endothelin-1	Potent vasoconstrictor and marker of endothelial dysfunction.	May reflect vascular maladaptation associated with absence of luteal vasoactive factors.	Remains under investigation in populations undergoing programmed FET.
Symmetric dimethylarginine	Endogenous inhibitor of NO synthase and marker of reduced NO bioavailability.	May indicate impaired endothelial function in CL-deficient pregnancies.	Has not yet been established for clinical use in programmed FET populations.
Inflammatory and oxidative stress markers	Reflect systemic vascular inflammation and endothelial injury involved in PE pathogenesis.	May provide additional information on maternal vascular maladaptation in programmed FET pregnancies.	Their clinical relevance in programmed FET populations remains to be established.

## Data Availability

The original data presented in the study are openly available in Medline/PubMed (https://pubmed.ncbi.nlm.nih.gov/; accessed on 25 July 2026), Scopus (https://www.elsevier.com/products/scopus; accessed on 25 July 2026), and Mendeley (https://www.mendeley.com/datasets; accessed on 25 July 2026).

## Abbreviations

The following abbreviations are used in this manuscript:

AC	arterial compliance
AGTR2	angiotensin II receptor type 2
ARTs	assisted reproductive technologies
BMI	body mass index
CL	corpus luteum
CPCs	circulating progenitor cells
EPCs	endothelial progenitor cells
FET	frozen embryo transfer
FGR	fetal growth restriction
GDM	gestational diabetes mellitus
HDPs	hypertensive disorders of pregnancy
LGA	large for gestational age
NO	nitric oxide
OHSS	ovarian hyperstimulation syndrome
PA	preeclampsia
PE	placental abruption
PLGF	placental growth factor
PP	placenta previa
RAAS	renin–angiotensin–aldosterone system
RHI	reactive hyperemia index
SGA	small forgestationalage
sFLt-1	soluble fms-like tyrosine kinase-1
VEGF	vascular endothelial growth factor

## References

[1] Roque M., Haahr T., Geber S., Esteves S.C., Humaidan P. (2019). Fresh versus Elective Frozen Embryo Transfer in IVF/ICSI Cycles: A Systematic Review and Meta-Analysis of Reproductive Outcomes. Hum. Reprod. Update.

[2] Shi Y., Sun Y., Hao C., Zhang H., Wei D., Zhang Y., Zhu Y., Deng X., Qi X., Li H. (2018). Transfer of Fresh versus Frozen Embryos in Ovulatory Women. N. Engl. J. Med..

[3] Wei D., Liu J.Y., Sun Y., Shi Y., Zhang B., Liu J.Q., Tan J., Liang X., Cao Y., Wang Z. (2019). Frozen versus Fresh Single Blastocyst Transfer in Ovulatory Women: A Multicentre, Randomised Controlled Trial. Lancet.

[4] Baker V.L., Iko I., Segars J. (2019). Is a Frozen Embryo Transfer in a Programmed Cycle Really the Best Option?. J. Assist. Reprod. Genet..

[5] Wang Z., Liu H., Song H., Li X., Jiang J., Sheng Y., Shi Y. (2020). Increased Risk of Pre-Eclampsia After Frozen-Thawed Embryo Transfer in Programming Cycles. Front. Med..

[6] Ghobara T., Gelbaya T.A., Ayeleke R.O. (2017). Cycle Regimens for Frozen-Thawed Embryo Transfer. Cochrane Database Syst. Rev..

[7] Von Versen-Hoÿnck F., Narasimhan P., Selamet Tierney E.S., Martinez N., Conrad K.P., Baker V.L., Winn V.D. (2019). Absent or Excessive Corpus Luteum Number Is Associated With Altered Maternal Vascular Health in Early Pregnancy. Hypertension.

[8] Von Versen-Hoÿnck F., Schaub A.M., Chi Y.Y., Chiu K.H., Liu J., Lingis M., Stan Williams R., Rhoton-Vlasak A., Nichols W.W., Fleischmann R.R. (2019). Increased Preeclampsia Risk and Reduced Aortic Compliance with In Vitro Fertilization Cycles in the Absence of a Corpus Luteum. Hypertension.

[9] Severino A.I., Póvoa A.M. (2021). Frozen Embryo Transfer and Preeclampsia Risk. J. Gynecol. Obstet. Hum. Reprod..

[10] Conrad K.P. (2011). Maternal Vasodilation in Pregnancy: The Emerging Role of Relaxin. Am. J. Physiol. Regul. Integr. Comp. Physiol..

[11] Conrad K.P., Baker V.L. (2013). Corpus Luteal Contribution to Maternal Pregnancy Physiology and Outcomes in Assisted Reproductive Technologies. Am. J. Physiol. Regul. Integr. Comp. Physiol..

[12] von Versen-Höynck F., Strauch N.K., Liu J., Chi Y.Y., Keller-Woods M., Conrad K.P., Baker V.L. (2019). Effect of Mode of Conception on Maternal Serum Relaxin, Creatinine, and Sodium Concentrations in an Infertile Population. Reprod. Sci..

[13] Pereira M.M., Mainigi M., Strauss J.F. (2021). Secretory Products of the Corpus Luteum and Preeclampsia. Hum. Reprod. Update.

[14] Debrah D.O., Novak J., Matthews J.E., Ramirez R.J., Shroff S.G., Conrad K.P. (2006). Relaxin Is Essential for Systemic Vasodilation and Increased Global Arterial Compliance during Early Pregnancy in Conscious Rats. Endocrinology.

[15] Conrad K.P. (2011). Emerging Role of Relaxin in the Maternal Adaptations to Normal Pregnancy: Implications for Preeclampsia. Semin. Nephrol..

[16] Smith M.C., Murdoch A.P., Danielson L.A., Conrad K.P., Davison J.M. (2006). Relaxin Has a Role in Establishing a Renal Response in Pregnancy. Fertil. Steril..

[17] Segal M.S., Sautina L., Li S., Diao Y.P., Agoulnik A.I., Kielczewski J., McGuane J.T., Grant M.B., Conrad K.P. (2012). Relaxin Increases Human Endothelial Progenitor Cell NO and Migration and Vasculogenesis in Mice. Blood.

[18] Von Versen-Höynck F., Brodowski L., Dechend R., Myerski A.C., Hubel C.A. (2014). Vitamin D Antagonizes Negative Effects of Preeclampsia on Fetal Endothelial Colony Forming Cell Number and Function. PLoS ONE.

[19] Muñoz-Hernandez R., Miranda M.L., Stiefel P., Lin R.Z., Praena-Fernández J.M., Dominguez-Simeon M.J., Villar J., Moreno-Luna R., Melero-Martin J.M. (2014). Decreased Level of Cord Blood Circulating Endothelial Colony-Forming Cells in Preeclampsia. Hypertension.

[20] Gumina D.L., Black C.P., Balasubramaniam V., Winn V.D., Baker C.D. (2017). Umbilical Cord Blood Circulating Progenitor Cells and Endothelial Colony-Forming Cells Are Decreased in Preeclampsia. Reprod. Sci..

[21] Wiegel R.E., Jan Danser A.H., Steegers-Theunissen R.P.M., Laven J.S.E., Willemsen S.P., Baker V.L., Steegers E.A.P., von Versen-Höynck F. (2020). Determinants of Maternal Renin-Angiotensin-Aldosterone-System Activation in Early Pregnancy: Insights From 2 Cohorts. J. Clin. Endocrinol. Metab..

[22] Carvalho L.N., Cristovam P.C., Passos C.S., Boim M.A. (2012). Mesangial Cells Cultured from Pregnant Rats Display Reduced Reactivity to Angiotensin II: The Role of Relaxin, Nitric Oxide and AT2 Receptor. Cell. Physiol. Biochem..

[23] Conrad K.P. (2016). G-Protein-Coupled Receptors as Potential Drug Candidates in Preeclampsia: Targeting the Relaxin/Insulin-like Family Peptide Receptor 1 for Treatment and Prevention. Hum. Reprod. Update.

[24] Sarwar M., Samuel C.S., Bathgate R.A., Stewart D.R., Summers R.J. (2015). Serelaxin-Mediated Signal Transduction in Human Vascular Cells: Bell-Shaped Concentration-Response Curves Reflect Differential Coupling to G Proteins. Br. J. Pharmacol..

[25] Conrad K.P., Petersen J.W., Chi Y.Y., Zhai X., Li M., Chiu K.H., Liu J., Lingis M.D., Williams R.S., Rhoton-Vlasak A. (2019). Maternal Cardiovascular Dysregulation during Early Pregnancy after In Vitro Fertilization Cycles in the Absence of a Corpus Luteum. Hypertension.

[26] Conrad K.P. (2020). Evidence for Corpus Luteal and Endometrial Origins of Adverse Pregnancy Outcomes in Women Conceiving with or Without Assisted Reproduction. Obstet. Gynecol. Clin. N. Am..

[27] Post Uiterweer E.D., Koster M.P.H., Jeyabalan A., Kuc S., Siljee J.E., Stewart D.R., Conrad K.P., Franx A. (2020). Circulating Pregnancy Hormone Relaxin as a First Trimester Biomarker for Preeclampsia. Pregnancy Hypertens..

[28] Bortoletto P., Prabhu M., Baker V.L. (2022). Association between Programmed Frozen Embryo Transfer and Hypertensive Disorders of Pregnancy. Fertil. Steril..

[29] Garovic V.D., Dechend R., Easterling T., Karumanchi S.A., Baird S.M.M., Magee L.A., Rana S., Vermunt J.V., August P. (2022). Hypertension in Pregnancy: Diagnosis, Blood Pressure Goals, and Pharmacotherapy: A Scientific Statement From the American Heart Association. Hypertension.

[30] Wen T., Schmidt C.N., Sobhani N.C., Guglielminotti J., Miller E.C., Sutton D., Lahtermaher Y., D’Alton M.E., Friedman A.M. (2022). Trends and Outcomes for Deliveries with Hypertensive Disorders of Pregnancy from 2000 to 2018: A Repeated Cross-Sectional Study. BJOG.

[31] Conrad K.P., von Versen-Höynck F., Baker V.L. (2022). Potential Role of the Corpus Luteum in Maternal Cardiovascular Adaptation to Pregnancy and Preeclampsia Risk. Am. J. Obstet. Gynecol..

[32] Vera-Ponce V.J., Loayza-Castro J.A., Ballena-Caicedo J., Valladolid-Sandoval L.A.M., Zuzunaga-Montoya F.E., Gutierrez De Carrillo C.I. (2025). Global Prevalence of Preeclampsia, Eclampsia, and HELLP Syndrome: A Systematic Review and Meta-Analysis. Front. Reprod. Health.

[33] Conrad K.P., von Versen-Höynck F., Baker V.L. (2024). Pathologic Maternal and Neonatal Outcomes Associated with Programmed Embryo Transfer: Potential Etiologies and Strategies for Prevention. J. Assist. Reprod. Genet..

[34] Koerts J.J., Voskamp L.W., Rousian M., Steegers-Theunissen R.P.M., Wiegel R.E. (2025). Impact of Corpus Luteum Number on Maternal Pregnancy and Birth Outcomes: The Rotterdam Periconception Cohort. Fertil. Steril..

[35] GinströmErnstad E., Wennerholm U.B., Khatibi A., Petzold M., Bergh C. (2019). Neonatal and Maternal Outcome after Frozen Embryo Transfer: Increased Risks in Programmed Cycles. Am. J. Obstet. Gynecol..

[36] Zaat T.R., Brink A.J., de Bruin J.P., Goddijn M., Broekmans F.J.M., Cohlen B.J., Macklon N.S., van Wely M., Groenewoud E.R., Mol F. (2021). Increased Obstetric and Neonatal Risks in Artificial Cycles for Frozen Embryo Transfers?. Reprod. Biomed. Online.

[37] Makhijani R., Bartels C., Godiwala P., Bartolucci A., Nulsen J., Grow D., Benadiva C., Engmann L. (2020). Maternal and Perinatal Outcomes in Programmed versus Natural Vitrified–Warmed Blastocyst Transfer Cycles. Reprod. Biomed. Online.

[38] Asserhøj L.L., Spangmose A.L., Aaris Henningsen A.K., Clausen T.D., Ziebe S., Jensen R.B., Pinborg A. (2021). Adverse Obstetric and Perinatal Outcomes in 1,136 Singleton Pregnancies Conceived after Programmed Frozen Embryo Transfer (FET) Compared with Natural Cycle FET. Fertil. Steril..

[39] Hu K.L., Zhang D., Li R. (2021). Endometrium Preparation and Perinatal Outcomes in Women Undergoing Single-Blastocyst Transfer in Frozen Cycles. Fertil. Steril..

[40] Zong L., Liu P., Zhou L., Wei D., Ding L., Qin Y. (2020). Increased Risk of Maternal and Neonatal Complications in Hormone Replacement Therapy Cycles in Frozen Embryo Transfer. Reprod. Biol. Endocrinol..

[41] Jing S., Li X.F., Zhang S., Gong F., Lu G., Lin G. (2019). Increased Pregnancy Complications Following Frozen-Thawed Embryo Transfer during an Artificial Cycle. J. Assist. Reprod. Genet..

[42] Saito K., Kuwahara A., Ishikawa T., Morisaki N., Miyado M., Miyado K., Fukami M., Miyasaka N., Ishihara O., Irahara M. (2019). Endometrial Preparation Methods for Frozen-Thawed Embryo Transfer Are Associated with Altered Risks of Hypertensive Disorders of Pregnancy, Placenta Accreta, and Gestational Diabetes Mellitus. Hum. Reprod..

[43] Dallagiovanna C., Cappellari M., D’Ambrosi F., Reschini M., Kordas K., Li Piani L., Filippi F., Somigliana E. (2022). Endometrial Preparation Does Not Affect the Risk of Hypertensive Disorders of Pregnancy in Low-Risk Women Undergoing Frozen Embryo Transfer. Gynecol. Endocrinol..

[44] Epelboin S., Labrosse J., De Mouzon J., Devaux A., Gervoise-Boyer M.J., Hesters L., Jonveaux P., Levy R., Sermondade N., Fauque P. (2023). Higher Risk of Pre-Eclampsia and Other Vascular Disorders with Artificial Cycle for Frozen-Thawed Embryo Transfer Compared to Ovulatory Cycle or to Fresh Embryo Transfer Following in Vitro Fertilization. Front. Endocrinol..

[45] Voskamp L.W., Koerts J., Wiegel R.E., Verdonk K., Danser A.H.J., Steegers-Theunissen R.P.M., Rousian M. (2025). Corpus Luteum Number and Maternal Circulatory Adaptation from Early Pregnancy Onwards: The Rotterdam Periconception Cohort (Predict Study). Hum. Reprod..

[46] Busnelli A., Schirripa I., Fedele F., Bulfoni A., Levi-Setti P.E. (2022). Obstetric and Perinatal Outcomes Following Programmed Compared to Natural Frozen-Thawed Embryo Transfer Cycles: A Systematic Review and Meta-Analysis. Hum. Reprod..

[47] Pohjonen E.M., Huhtala H., Erkinaro T., Lehto J., Pellas E., Vilmi-Kerälä T., Laivuori H., Ahinko K. (2024). Risk Assessment of Hypertensive Disorders of Pregnancy and Other Adverse Pregnancy Outcomes after Frozen Embryo Transfers Following an Artificial Cycle: A Retrospective Cohort Study. Int. J. Gynaecol. Obstet..

[48] Takeshima K., Ezoe K., Onogi S., Kawasaki N., Hayashi H., Kuroda T., Kato K. (2022). Endometrial Preparation and Maternal and Obstetrical Outcomes after Frozen Blastocyst Transfer. AJOG Glob. Rep..

[49] Sites C.K., Wilson D., Barsky M., Bernson D., Bernstein I.M., Boulet S., Zhang Y. (2017). Embryo Cryopreservation and Preeclampsia Risk. Fertil. Steril..

[50] Wong K.M., van Wely M., Mol F., Repping S., Mastenbroek S. (2017). Fresh versus Frozen Embryo Transfers in Assisted Reproduction. Cochrane Database Syst. Rev..

[51] Carosso A.R., Brunod N., Filippini C., Revelli A., Evangelisti B., Cosma S., Borella F., Canosa S., Benedetto C., Gennarelli G. (2023). Reproductive and Obstetric Outcomes Following a Natural Cycle vs. Artificial Endometrial Preparation for Frozen-Thawed Embryo Transfer: A Retrospective Cohort Study. J. Clin. Med..

[52] Wang Z., Zhang Y., Shang X., Miao R., Yin M., Yang H., Yu Y., Wei D. (2023). The Likelihood of a Healthy Live Birth after Frozen Embryo Transfer with Endometrium Prepared by Natural Ovulation Regimen vs Programmed Regimen: A Propensity-Score Matching Study. AJOG Glob. Rep..

[53] Wang X., Li Y., Tan H., Cai S., Ma S., Peng Y., Guo H., Li X., Tang Y., Zhang S. (2023). Letrozole-Stimulated Endometrial Preparation Protocol Is a Superior Alternative to Hormone Replacement Treatment for Frozen Embryo Transfer in Women with Polycystic Ovary Syndrome, a Cohort Study. Reprod. Biol. Endocrinol..

[54] Hu X., Liao Z., Li J., Zhou Y., Guo Y., Qian K. (2023). Young Women Were Associated with Higher Risk of Hypertensive Disorders of Pregnancy and Cesarean Section from Hormone Replaced Cycles in Frozen-Thawed Embryo Transfer: A Retrospective Study of 5316 Singleton Deliveries. Front. Endocrinol..

[55] Wang F., Wang Q., Song Y., Ding J., Li H., Meng Q. (2023). Programmed Frozen Embryo Transfer Cycles Are Associated with a Higher Risk of Abnormal Placental Development: A Retrospective Cohort Study of Singleton Live Births. Front. Endocrinol..

[56] Fan L., Li N., Liu X., Li X., Cai H., Pan D., Wang T., Shi W., Qu P., Shi J. (2023). Hormone Replacement Treatment Regimen Is Associated with a Higher Risk of Hypertensive Disorders of Pregnancy in Women Undergoing Frozen-Thawed Embryo Transfer. Front. Endocrinol..

[57] Wang Y., Chen Q., Wang Y. (2022). Effects of Different Endometrial Preparation Regimens during IVF on Incidence of Ischemic Placental Disease for FET Cycles. J. Clin. Med..

[58] Pape J., Levy J., von Wolff M. (2023). Hormone Replacement Cycles Are Associated with a Higher Risk of Hypertensive Disorders: Retrospective Cohort Study in Singleton and Twin Pregnancies. BJOG.

[59] Waschkies F., Kroning L., Schill T., Chandra A., Schippert C., Töpfer D., Ziert Y., von Versen-Höynck F. (2021). Pregnancy Outcomes After Frozen-Thawed Embryo Transfer in the Absence of a Corpus Luteum. Front. Med..

[60] Zhao Z., Chen Y., Deng H., Huang L., Lu D., Shen X., Xu L. (2023). The Influence of Embryo Stage on Obstetric Complications and Perinatal Outcomes Following Programmed Compared to Natural Frozen-Thawed Embryo Transfer Cycles: A Systematic Review and Meta-Analysis. Front. Endocrinol..

[61] Gasse C., Boutin A., Coté M., Chaillet N., Bujold E., Demers S. (2018). First-Trimester Mean Arterial Blood Pressure and the Risk of Preeclampsia: The Great Obstetrical Syndromes (GOS) Study. Pregnancy Hypertens..

[62] Meeme A., Buga G.A.B., Mammen M., Namugowa A. (2017). Endothelial Dysfunction and Arterial Stiffness in Pre-Eclampsia Demonstrated by the EndoPAT Method. Cardiovasc. J. Afr..

[63] Weissgerber T.L., Milic N.M., Milin-Lazovic J.S., Garovic V.D. (2016). Impaired Flow-Mediated Dilation Before, During, and after Preeclampsia: A Systematic Review and Meta-Analysis. Hypertension.

[64] Gu F., Wu Y., Tan M., Hu R., Chen Y., Li X., Lin B., Duan Y., Zhou C., Li P. (2023). Programmed Frozen Embryo Transfer Cycle Increased Risk of Hypertensive Disorders of Pregnancy: A Multicenter Cohort Study in Ovulatory Women. Am. J. Obstet. Gynecol. MFM.

[65] Rodriguez E., Jackson F., Blanco-Breindel M., Blitz M., Goldman R., Weinberg C. (2025). Abstract 4365095: Not So Relaxin: Preeclampsia Risk After Frozen Embryo Transfer with Absent Corpus Luteum. Circulation.

[66] Levine R.J., Maynard S.E., Qian C., Lim K.H., England L.J., Yu K.F., Schisterman E.F., Thadhani R., Sachs B.P., Epstein F.H. (2004). Circulating angiogenic factors and the risk of preeclampsia. N. Engl. J. Med..

[67] Zeisler H., Llurba E., Chantraine F., Vatish M., Staff A.C., Sennström M., Olovsson M., Brennecke S.P., Stepan H., Allegranza D. (2016). Predictive Value of the sFlt-1:PlGF Ratio in Women with Suspected Preeclampsia. N. Engl. J. Med..

[68] Conrad K.P., von Versen-Höynck F., Baker V.L. (2022). Risk of preeclampsia in artificial frozen embryo transfer as a result of insufficient corpus luteum hormone levels: A response. Am. J. Obstet. Gynecol..

[69] Sherwood O.D. (2004). Relaxin’s physiological roles and other diverse actions. Endocr. Rev..

[70] Böger R.H. (2006). Asymmetric dimethylarginine (ADMA): A novel risk marker in cardiovascular medicine and beyond. Ann. Med..

[71] Sanghavi M., Rutherford J.D. (2014). Cardiovascular physiology of pregnancy. Circulation.

[72] Melchiorre K., Sutherland G.R., Baltabaeva A., Liberati M., Thilaganathan B. (2011). Maternal cardiac dysfunction and remodeling in women with preeclampsia at term. Hypertension.

[73] Melchiorre K., Sharma R., Thilaganathan B. (2014). Cardiovascular implications in preeclampsia: An overview. Circulation.

[74] Zaat T.R., Kostova E.B., Korsen P., Showell M.G., Mol F., Van Wely M. (2023). Obstetric and Neonatal Outcomes after Natural versus Artificial Cycle Frozen Embryo Transfer and the Role of Luteal Phase Support: A Systematic Review and Meta-Analysis. Hum. Reprod. Update.

